# Spatial organization to facilitate action

**DOI:** 10.1371/journal.pone.0216342

**Published:** 2019-05-10

**Authors:** Grayden J. F. Solman, Alan Kingstone

**Affiliations:** 1 Psychology, University of Hawai’i at Mānoa, Honolulu, HI, United States of America; 2 Psychology, University of British Columbia, Vancouver, BC, Canada; University of Plymouth, UNITED KINGDOM

## Abstract

Humans exert a great deal of control over our local environments–selecting and arranging the many objects around us on the basis of conflicting task-demands, aesthetic preferences, and habitual convenience. Because routine behaviour necessitates that we regularly find and access these objects, the particular arrangements we choose can influence the likelihood and difficulty of engaging in different tasks and actions. Despite this importance, relatively little research has directly examined human organizational behaviours and tendencies. Here we investigate how objects in a computer-based search task are freely and dynamically arranged by participants over time, while manipulating the statistics of the target sequence. We report common organizational behaviours including reduction of distance between targets as well as separation of target subsets with high community. However, the extent of these behaviours and their relationship to individual differences in performance varies as a function of the target sequence structure. In particular, tasks composed of a larger number of smaller groups of targets lead to better organizational and performance outcomes than tasks composed of fewer larger groups.

## Introduction

Though many animals actively shape the environment to suit their needs (cf., ecosystem engineers [1,2] and niche-construction [3]), humans do so to an unrivaled extent, producing objects and structures far greater in scale, number, and diversity than any other creature. On an individual basis, we make ongoing decisions throughout our daily lives concerning which objects to obtain, which to discard, and where to place these various objects in relation to the environment, to each other, and to ourselves. These decisions necessarily shape our behaviour and decision making, influencing the relative ease of perceptual and physical access, and thereby shaping our evaluations of which behaviours are possible, and which behaviours are convenient or practical as we pursue our higher goals.

Many experiments have investigated object perception in isolation and in the context of meaningful scenes [4–7], and further how each of these momentary perceptions can affect subsequent decisions and actions [8,9]. What has rarely been investigated, however, is the fundamental question of how these perceptions, decisions, and actions loop back to directly influence future perceptions, decisions, and actions in an ongoing cycle [10]. Although the deliberate shaping and organization of our environments forms the backbone of this cycle and is central to naturalistic human behaviour, the issue has received surprisingly little attention in psychological research. A number of observational and theoretical studies have provided many of the key insights and frameworks for thought on the subject. Broadly, organizational actions have been characterized as being either ‘epistemic’ or ‘pragmatic’ [11]. Epistemic actions are those whose purpose is to reduce cognitive burdens by arranging the environment in a way that simplifies or obviates information-seeking. Pragmatic actions, in contrast, are those *“whose primary function is to bring the agent closer to his or her physical goal*,*”* with typical discussions of planning concerned with how best to sequence pragmatic actions to *“serve as a path from initial to goal state”* [11]. It should be noted that these are not mutually exclusive categories–many actions can be reasonably considered to advance both cognitive and pragmatic aims (as in the case of laying out ingredients prior to cooking). However, there are also circumstances where these aims can be at odds. For example, emptying out a drawer and spreading out its contents in order to find a particular object facilitates the search process (epistemic organization), but results in a disordered state where extra work is necessary to replace everything in the drawer. The labels ‘epistemic’ and ‘pragmatic’, then, are most profitably applied as independent characteristics–not as a dichotomy or a spectrum.

In addition to the distinction between epistemic and pragmatic actions, Kirsh [12] identifies the importance of timescale in discussions of organization, using the example of a short order cook who maintains his kitchen (long term), prepares tools for a particular order (medium term), and adaptively arranges and rearranges those tools in the process of completing the order (short term).

Discussion and research on the arrangement of space has largely focused on epistemic actions that reduce cognitive load, typically at the medium or short timescale. From careful observational work, Kirsh [12] suggests that spatial organization behaviours serve the function of streamlining the environment by limiting the number of ‘choice-points’ where an individual must decide upon the next action. In this way, behaviour can proceed reflexively, under the guidance of environmental cues, sparing the cognitive resources that would otherwise be necessary for planning (see also, [13,14]). Indeed, there is even evidence that greater neural resources are devoted to representations of objects at choice points [15]. Similarly, in an observational and interview-based study of personal offices, it was reported that many organizational decisions are made not only to facilitate task completion, but also to serve as physical reminders (e.g., the use of sticky notes) of important or unfinished tasks [16]. Empirical work has also largely focused on epistemic actions, showing that individuals prefer to manipulate the world or their relation to the world rather than simulating those manipulations mentally [11,17–20], and that performance on a range of cognitive tasks is improved when participants are given the opportunity to interact with and shape the task environment (e.g., [21–24]).

Comparatively little work has addressed pragmatic organization–decisions intended not to reduce *cognitive* demands, but to make action itself easier (e.g., by reducing the time or effort required to access or interact with an object). Rarer still is the discussion of *long term* organization. The conjunction of these two categories–long term pragmatic organization–exhibits two particularly important characteristics: 1) these organizational decisions are probabilistic; they are driven by *expected* future demands rather than an explicit immediate goal, and 2) these decisions concern *multiple* goals, reflecting the need to balance and coordinate different sets of demands in contrast to a single task-specific set. Indeed, where much of the prior literature has focused on the value of short- and medium-term epistemic actions in *limiting* behavioral choices and outcomes, we suggest that long-term pragmatic decisions often serve instead to *increase* the range of available actions to support flexibility for future tasks that may be conflicting or underspecified. This type of organizational behaviour is arguably of central importance to understanding our relationship to space, as it provides the stable ‘background’ in which individual tasks must unfold (cf. ‘environment stabilization’ [25]). Long term pragmatic behaviours describe both the initial set-up of a space (e.g., moving in to a new home), as well as the gradual ongoing adjustments and accommodations that continue to shape a space over time and over the course of myriad different activities.

A small handful of recent studies have sought to explore pragmatic organizational behaviours on shorter timescales. Zhu and Risko [26] had participants complete a symbol-copying task using two pens (differentiated by colour) placed in separate pen holders, with one pen holder farther away than the other. They found that, when one colour of pen was used more often than the other, and when the distance between the pen holders was relatively large (45 cm), participants would usually reconfigure the placement of the pens so that the more frequently used pen was located in the closer holder. These results show that even in a simple two-object environment, individuals are sensitive to access demands, and will actively reorganize their space to minimize those costs.

In another recent study of pragmatic action, we introduced a computer-based task that combined search, item access, and decision-making while providing participants with full and ongoing control over the configuration of items in the display [27]. As the present work follows closely from this task, we take a moment here to describe it in detail. Participants navigated through a large space via a click-and-drag panning interface, with the aim of locating and interacting with individual target items. The task was designed to incorporate several key principles of spatially embedded action:

1. At any given moment, the agent has a specific position in space, with corollaries: i) distant objects are more difficult to discriminate than nearby ones, and ii) only objects within a limited area (i.e., within ‘reach’) can be interacted with;
2. In order to obtain a target (i.e., ‘use’ an object), it is necessary to interact with the object–not simply visually locate it; and
3. It is at the agent’s discretion where to replace an object in the environment once they are done with it (i.e., the agent is free to reorganize objects in the environment).

In two experiments using this task, Solman and Kingstone [27] manipulated the sequence in which targets were presented in order to examine the relationship between organizational outcomes and frequency of target use. A complete description of these target sequences is beyond the current scope, but it is important to note that participants had a choice between two or more different targets at any given time. In the first experiment, sequences were essentially random, so that the only meaningful distinction was between targets used more or less frequently over time. In this initial experiment, we reported similar results to those of Zhu and Risko [26]–that over time, more frequently used items tended to be centralized in the virtual space, while less frequently used items were moved to the periphery. In the second experiment, the target sequence was engineered so that, in set successive periods of 12-target runs, participants made repeated forced choices between two distinct subsets of items to be located and accessed (i.e., they could choose to obtain 12 targets from one subset or the other). In this case, we found that items from the chosen subset were selectively centralized and clustered, and that, on an individual basis, the tendency to demonstrate this organizational pattern was correlated with performance on the task.

While these preliminary studies of pragmatic action have exposed important and reliable organizational tendencies, they have been limited to single-task settings (i.e., with a single, fixed set of target relationships). As one of the primary research goals in the social sciences in general, and in cognitive science in particular, is to test the boundary conditions of the principles exposed in prior work, it remains to be seen how the principles we have previously uncovered generalize to more realistic long-term settings where multiple tasks are undertaken within the same environment. The purpose of the present experiments is to address this issue. To this end, we adapt an interactive search task [27] to simulate long-term, multiple-task conditions by manipulating the target sequence to cycle between several distinct subsets of items. This addresses a critical limitation in our prior work. Specifically, because participants were always able to choose between targets (and additionally, in Experiment 2, between target subsets), there was never a requirement to balance competing demands, or, equivalently, to delineate and balance subtasks within a single setting.

To address this, in the present study we define target sequences with high *community* in their item-item transitions–i.e., with subsets of items that are frequently used in succession with each other, and only rarely used in succession with items from other subsets. This type of high-community structure can be readily observed in everyday situations (e.g., objects from the kitchen are consistently used one after another, but rarely used in sequence with objects from the bedroom). However, it should be noted that the term ‘community’–and our use of it in the present work–refers only to the statistical structure of a set of relations, and not to the potential semantic groupings that might be associated. In the present work, we evaluate two community structures–one with two subsets of twelve items each (‘2x12’ condition), and one with four subsets of six items each (‘4x6’ condition). In this way, both target sequences include the same total number of distinct items, and have uniform long-run frequencies across individual targets, but differ markedly in their *sequential* properties. Our analyses focus on how organizational decisions–for equally-sized collections of objects–may differ on the basis of these different temporal structures, and to what extent these decisions support performance.

It is important to note that the current approach is suited to studying the *emergence* of organization in the absence of explicit task knowledge or semantic priors. In other words, we are not examining the type of long-term strategic organization that might be observed when moving into a new home. Instead, the present task is more closely analogous to the organizational adjustments that come from living in a space and engaging repeatedly with common tasks, gradually learning their requirements and how best to structure the environment to facilitate them. This is, in the context of a single-session laboratory study, a necessary restriction.

Although prior work in this area is limited, we can make some predictions about the expected behaviors. First, we have previously reported that individuals–particularly those with high performance–show a tendency to contract the distances between task-relevant objects. Consequently, we can expect a similar clustering in the present experiment. Less certain is whether or not participants will show a further sensitivity to the different subsets present within the target streams. If they are sensitive to these properties, individuals might produce distinct clusters for each subset–effectively minimizing the expected distance between successive targets. Indeed, partitioning space into distinct task-specific regions is typical of naturalistic environments–the kitchen and the bedroom are not merely conceptual sets, they are tangible and distinct spatial regions. The likelihood of adopting this kind of partitioning strategy, however, may depend on the ease of identifying the boundaries between subsets–i.e., the difference between within-set transition rates and between-set transition rates. For this reason, we may expect partitioning to occur more readily in the 4x6 condition, where this difference in transition rates is stronger than in the 2x12 condition.

A secondary question for the present work concerns *how* organization arises. In particular, we ask whether individuals organize their search environments in a punctuated or incremental way–i.e., rearranging many items all at once, or instead adjusting single items here and there throughout the task. The relative prevalence of these two approaches will provide insight into the extent to which this class of organization is global and deliberate (resulting in infrequent large-scale rearrangements), or more local and emergent (arising from ongoing moment-to-moment decisions about recent and upcoming needs). The relative prevalence and effectiveness of these strategies may provide insights into metacognition and forecasting of future needs and their expected costs, as well as informing how responsive individuals would be to changes in task demands. Although global organizing might be expected to represent the most overtly strategic approach, the reliable tendency towards cognitive offloading suggests that individuals may prefer a less cognitively-demanding approach involving incremental or emergent changes restricted to temporally-local demands. Conversely, the ongoing disruption to memory formation expected from continual small changes to the display may be more costly in the long run than single widespread rearrangements, favoring global reorganization.

## Methods

### Conceptual motivations

There are a number of requirements for a task to support organizational behaviors, and in the interests of contextualizing the detailed methods to follow, we first lay out the particular needs that these methods were designed to address. In Solman and Kingstone [27], we identified several properties of naturalistic tasks that we wished to capture in our methodology. First, we note that naturalistic tasks can, with generality, be framed in terms of the *sequential access* of objects (whether these are tools, resources, information sources, etc.), and the particular sequence required for a given goal is usually transparent to the actor–i.e., upcoming demands are known in advance. Second, naturalistic tasks are *spatially embedded*; objects are distributed throughout the environment, and the relative positions of objects have implications for behavior–more distant objects are both more difficult to perceive and more difficult to access. Finally, and of particular importance for the present work, the majority of the objects we use in naturalistic tasks must be physically accessed–i.e., they must be ‘*in-hand*’ and not merely inspected visually. To use an object, we need to be close to it, we need to pick it up, and when we are finished we must put it down again. The methods that follow were designed to address these requirements in a way that was practical, manipulable, and analytically tractable.

### Qualitative overview

**Fig 1:**
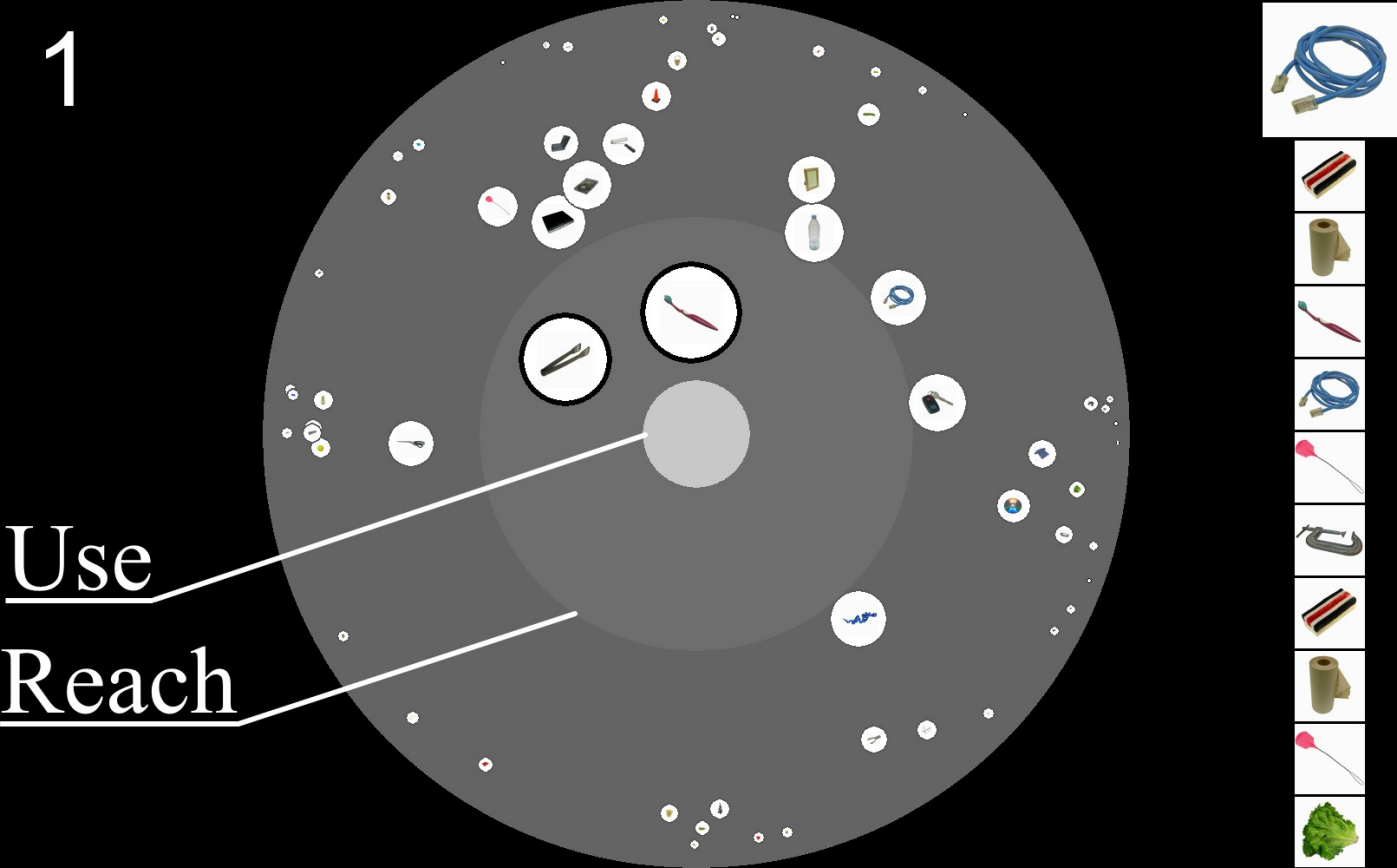
Example display, with circular search space in the centre, target sequence on the right (the slightly larger item at the top of the list is the immediate target), and current score (‘1’ here) displayed in the upper left. Search space: items are presented on white circles, with size scaled as a function of distance in the display. To use an item, the agent must move in the space (by clicking and dragging) so that the item falls within the outer grey region (the ‘Reach’ area), then select the item and drag it onto the middle pale grey region (the ‘Use’ area). See vimeo.com/239549059 for a video example.

**Fig 2:**
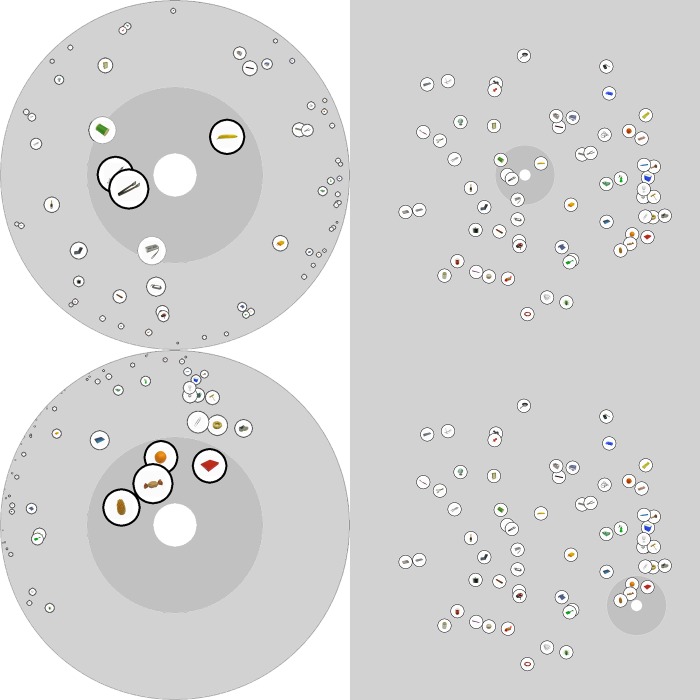
Example displays showing the hyperbolic mapping used in the Action Space. On the right, items are displayed in the underlying flat coordinate space, and on the left, the corresponding participant view is shown. Note that regardless of where the participant is located in the flat coordinate space (central in top example, lower right in bottom example), this position is mapped to the centre of the visible display. Items in the display are presented in their relative radial positions, with size and radial distance scaled as a function of absolute distance in the flat coordinate space.

The complete details of the paradigm are laid out below, but given the requisite complexity of this highly interactive task, it is helpful to begin with a qualitative impression of how the task appears and unfolds from the perspective of the participant. A sample display is presented in Fig 1. The primary component of the display is a circular field of randomly arranged object images differing in size–larger towards the middle, and smaller towards the edges of the field. Using the mouse to click and drag across the field, the participant is able to adjust their perspective. Much like computer-based panning interfaces (e.g., while viewing maps or large images), nearby information remains centred in the display at all times. In appearance, these adjustments are much like rotating a globe–the central details are larger and more prominent, and grow smaller as rotation pulls them towards the periphery. By rotating a globe, details of one region can be emphasized at the expense of others, much in the way that moving physically through space causes nearby objects to be more discriminable at the expense of objects farther away. In the present task, this globe-like appearance is misleading–the underlying space is actually not a sphere, but instead an infinite plane condensed to a circle via a hyperbolic map (Fig 2)–but a globe provides a qualitatively similar and more intuitive analogy to help the reader’s visualization.

Within this space, the participant’s goal is simple, for each target in a given sequence, they must locate that target, adjust the display so that it is close to the centre, then click and drag the target onto the very centre. The reader might imagine being asked to tap on the location of a particular city on a globe. They would first need to rotate the globe so that they could both see and reach the city. In same way, participants needed to click-and-drag to navigate through the virtual space until the target item was ‘within reach’ of the centre, then actively move that item onto the centre to register target acquisition. A target must be located, within reach, and directly manipulated–our key conceptual requirements.

The last, and most important feature, is that participants were free to move any item (targets and distractors alike) at any time to any position within the space. These movements were subject to the same requirements as target access–to be moved, an object must first be within-reach, and must be actively manipulated–but otherwise were unrestricted. In this way, participants were free to arrange their environment however they chose–bringing particular items closer together or farther apart at their discretion. These organizational decisions would then necessarily influence the relative ease of subsequent location and access behaviours.

### Participants

100 undergraduate students (72 F, 28 M) from the University of British Columbia participated for course credit and a small performance-contingent food reward (salty or sweet snacks, carbonated drinks, of the participant’s choosing). Informed consent was obtained from all participants, and all experimental procedures and protocols were reviewed and approved by the University of British Columbia Behavioral Research Ethics Board (H10-00527). Fifty participants (41 F, 9 M) completed the 2x12 subset version of the task, and fifty participants (31 F, 19 M) completed the 4x6 subset version of the task (see procedures). Expected effect sizes are difficult to reliably estimate, as little prior work has examined related questions or measures. Where comparable tests exist, effect sizes are moderate–e.g., area reduction over time, as measured by Solman and Kingstone [27], had an effect size of 𝑓^2^ ~ .154. Given the similarity in experimental procedures, we anticipate similar effect sizes in the present work. Consequently, for a power of 0.95, a minimum of 42 participants per condition would be required. All participants reported normal or corrected-to-normal visual acuity, and normal color vision. One participant in the 4x6 condition obtained only 59 targets, and so was excluded (critically, this performance is so low that the participant did not even encounter every target in the task).

### Displays

A sample display is shown in Fig 1 (A video of the experiment can be viewed at: vimeo.com/239549059). In the top left, the current score on the task is displayed–a running count of the number of targets the participant has obtained. In the figure, one target has been located and accessed thus far. On the right, the current target (topmost and largest) is displayed along with the following ten targets (satisfying the requirements of sequential access and foreknowledge). As each target is located and accessed, the list shifts upwards and a new item is inserted at the bottom. In the example figure, when the blue cable has been located and accessed, the blackboard eraser will shift up to become the current target, followed by the paper towel roll, and so on, and a new item will be inserted underneath the lettuce at the bottom of the list. The circular region in the centre of the display is where participants search for, reorganize, and access target items, as described below.

The search space, designed to satisfy spatial dependency and in-hand use requirements, comprises three concentric circular regions, which can be conceptualized, from the outside in, as the full environment, a peripersonal ‘reach’ space, and the body. Interaction with these regions, and their relation to the task, is described in the Procedures section below. The full search space had a radius of 500 pixels, a reach radius of ½ the full radius, and a ‘body’ radius of ⅛ the full radius. The body was displayed in light grey (200), the reach area in medium grey (110), and the remaining area in a slightly darker grey (100).

For each participant, a random set of 64 object images was drawn from a subset of the Bank of Standardized Stimuli [28]. By randomizing item identities across participants, we ensure that incidental semantic relationships that might exist between items will not bias the results in any systemic fashion. Within the search space, one instance of each of the 64 items was present, displayed on a circular white disc. The initial configuration of item positions was randomly generated in a ‘raw’ coordinate space centered at (0,0) and inside the circle with radius = 1.0 (arbitrary units). The participant’s position in the space was initially at the origin (0,0).

To simulate a large environment within a limited two-dimensional display, we use a hyperbolic mapping to scale the visible size of each item by its distance from the participant’s position in the raw coordinate space. This mapping was preferred over a ‘flat’ display with panning (e.g., in common online mapping software, or when viewing a zoomed in image on a small computer screen), because it better captures the perceptual nature of three-dimensional space; objects in the real world are not seen at a constant size across distance, and do not simply vanish when they pass an arbitrary threshold distance like the edge of a screen. Instead, the visual angle of an object smoothly and consistently decreases with distance from the viewer, and does so indefinitely until the point that it is smaller than can be perceptually resolved. In the same way, items in a two-dimensional hyperbolic mapping grow ever smaller as they move towards the edge of the space, but never disappear. The parameters for the mapping were: base (*h*) = 1.5, and characteristic distance (*c*) = 0.12 (in coordinate space units). With these parameters, item size (*s*) and onscreen distance from the search space centre (*d)*, scaled with coordinate space distance (d*) as follows:

$$s=\left(maximum\;size\right)•h^{‐(d*/c)}$$

$$d=\left(search\;space\;radius\right)•\left(1.0‐h^{‐(d*/c)}\right)$$

Maximum item size was 100 pixels at *d* = 0.0, and minimum item size was held at 2 pixels, so that arbitrarily distant items were still visible (albeit not discriminable). Items fully within the reach radius (only these items could be interacted with, see procedures below) were highlighted with a thicker black border. Example display configurations (left side) are shown alongside their raw coordinate space configurations (right side; not displayed to the participant) in Fig 2. Note that as the participant moves from the centre of the coordinate space (top right panel in the figure) to the lower right corner of the coordinate space (bottom right panel in the figure), the *visible* display changes so that items close to the participant are large and near the centre, while items far from the participant are small and near the edge–however, the arrangement of objects in the raw coordinate space does not change. This form of display mapping mirrors the real world characteristics of large environments–as we move through the world, objects near to us become visually large and easier to discriminate, while objects farther away become visually small and harder to discriminate.

### Procedure

Each participant was given a random set of 64 items, and began the task with a random initial configuration of items in the search space. Target sequences were generated so as to have community structure with either two subsets of twelve items each (2x12 condition) or four subsets of six items each (4x6 condition). As a result, 24 items in the display were possible targets, while the remaining 40 served as distractors. The target sequence was constructed hierarchically, first by generating a list of subsets (simply alternating for the 2x12 condition, and pseudorandomized for the 4x6 condition so that subsets could not repeat back to back, and ensuring equal representation of each subset), then by generating a random sequence of between 8 and 32 targets (without back-to-back repetition) for each subset. Following 250 targets, sequences and subsets were randomly regenerated in order to investigate how individuals adjust to changing demands. However, not all participants reached this transition point, so for the present work we evaluate organization only over the first 250 targets.

Participants interacted with the display using the mouse. By clicking and dragging, they could change their position in the raw coordinate space, thereby changing which items were in reach and which were merely visible (recall Fig 2). Note that the ‘body’ region always remains central in the display, regardless of position in the coordinate space–i.e., the task uses an egocentric reference frame. Following Solman and Kingstone [27], the ‘body’ region was imbued with a semblance of physicality, such that it could not move over top of items in the display, but would instead push them out of the way. Similarly, an item ‘dropped’ on top of the body would be pushed outward to land in the nearest non-body region of the space. In other words, although items could overlap each other, they could not overlap with the central ‘body’ region.

In order to move a given item within the space, the item first had to be within the reach area, at which point it could be selected with the mouse and moved independently within the reach area, to be released in whatever position the participant wished. An item could be moved over greater distances (i.e., greater than the reach radius), by dragging it to the edge of the reach area, whereupon the participant’s location within the coordinate space would also move in that direction. This is analogous to the fact that, to place an object outside of your arm’s reach, it is necessary to also move yourself through space. Note that both targets and distractors could be moved freely in this way.

In order to obtain a target, a participant had to locate the item, move through the space to bring the item within reach, and then select and drag the item on top of the ‘body’ region. Successful target acquisition was signaled by a ‘click’ sound, which occurred at the instant of overlap between the item and the ‘body’ region, and triggered an update to both the target sequence and the score counter. The relevant item could either be deliberately replaced, or simply released–in which case the ‘body’ region would repel the item to the nearest valid position (see above). One point was awarded for each target obtained.

Participants were instructed via a brief demonstration–we appreciate that the task, described in static written language, may seem complex. However, participants found the task intuitive and straightforward, and had no trouble understanding the interface with only a brief demonstration. The researcher would show the participant how to use the mouse to move through the environment, to select and move items, and to obtain a target item. The experiment was then reset (with new items, configuration, and target sequence), and the participant was instructed to maximize the number of targets obtained over an allotted time of ~45 minutes. At the end of the experiment, points could be exchanged for small food rewards (salty or sweet snacks, carbonated drinks). Importantly, no further instructions were provided: organization was neither encouraged nor mentioned, nor was there any indication that the sequence of targets was structured. All aspects of task performance were at the participant’s sole discretion, with free movement through space and free movement of individual items within the space. Participant actions and the states of the action space and goal space were continuously monitored throughout the experiment.

### Apparatus

The experiment was written and executed in Python using the pygame module, and run on an Apple mini, with OS X 10.6.4 and a 2.4GHz Intel Core 2 Duo processor. The stimulus displays were presented on a 24” Dell Acer V243H monitor at a resolution of 1920 by 1080. Seating distance was not rigidly controlled, but was approximately 60 cm.

## Results

### Analytic approach

Following prior work, we examine organization changes by comparing the randomly-generated initial configuration of items to the participant-generated terminal configuration of items. We first examine overall changes from initial to terminal configurations, then examine how organizational measures relate to individual differences in performance. Finally, we explore how organization emerges at a finer temporal scale by examining individual rearrangement events and their influence on organizational outcomes.

Our primary variables of interest stem from the predicted organizational outcomes of contraction and separation. While there are numerous functionally-equivalent measures of clustering, we have previously used the *convex hull* to capture the overall area ‘occupied’ by a given set of items [27]. This is the smallest possible convex region that covers all the relevant points, and is uniquely determined. A common analogy for the process is to imagine placing pegs at each location of interest, then wrapping an elastic band around them. The form that the band relaxes to is the convex hull for those points. While there are several alternative and roughly equivalent (i.e., highly correlated) measures, such as the mean distance to centroid, or the mean inter-item distance, the convex hull has the advantage that it also provides a ready and transparent measure of ‘overlap’ between sets. Consequently, the convex hull provides a single framework for measuring both contraction (a reduction in hull area) and separation (a reduction in overlap between hulls).

### Hull area

**Fig 3:**
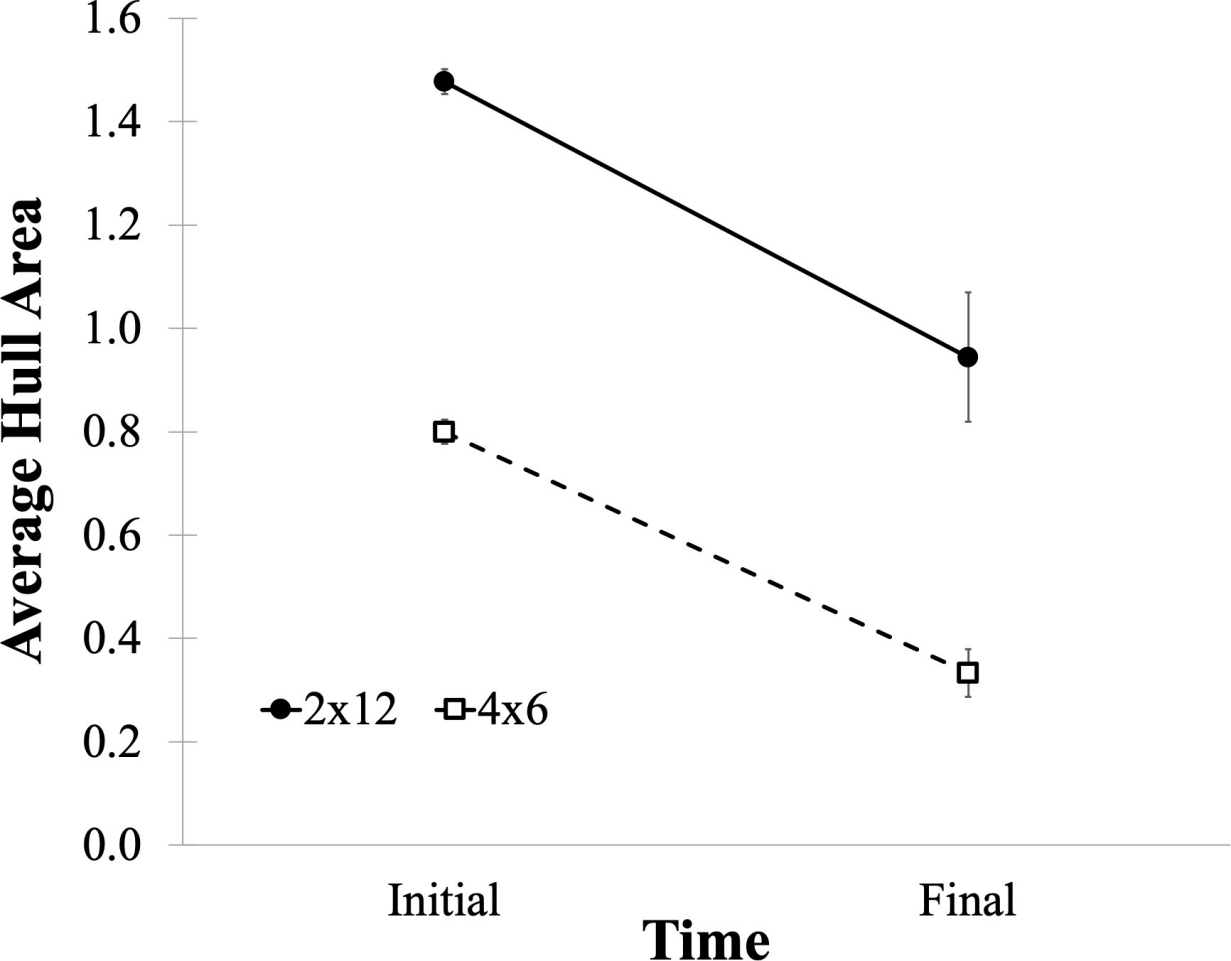
Average hull areas for the 2x12 (solid) and 4x6 (dashed) conditions, plotted for Initial and Final configurations, with error bars depicting one standard error of the mean.

To measure the extent to which items within a set have been brought closer together in space, we compute convex hulls for each item set, and evaluate the average area of these hulls at the beginning and end of the test period. A smaller area indicates that the items in the set have been brought closer together. Hull areas are plotted in Fig 3, and were analyzed with a Time (Initial, Final) by Condition (2x12, 4x6) ANOVA. Note that we expect hulls to be larger in the 2x12 condition than in the 4x6 condition, as each set contains twice as many items, and indeed this effect was significant, F(1,97) = 87.2, MSE = .235, p < .0001. We also find significantly smaller areas in the Final as opposed to the Initial configuration, F(1,97) = 51.3, MSE = .240, p < .0001, indicating that bringing items closer together over time was a consistent strategy for participants in this task, in keeping with past results. The interaction, however, was not significant, F(1,97) = 0.055, p = .633. In both conditions, the average area of the set hulls was reduced by the same amount–the 12-item sets in the 2x12 condition started larger and remained larger than the 6-item sets in the 4x6 condition. We can conclude, then, that area reduction was a common organizational approach in both conditions.

### Overlap

**Fig 4:**
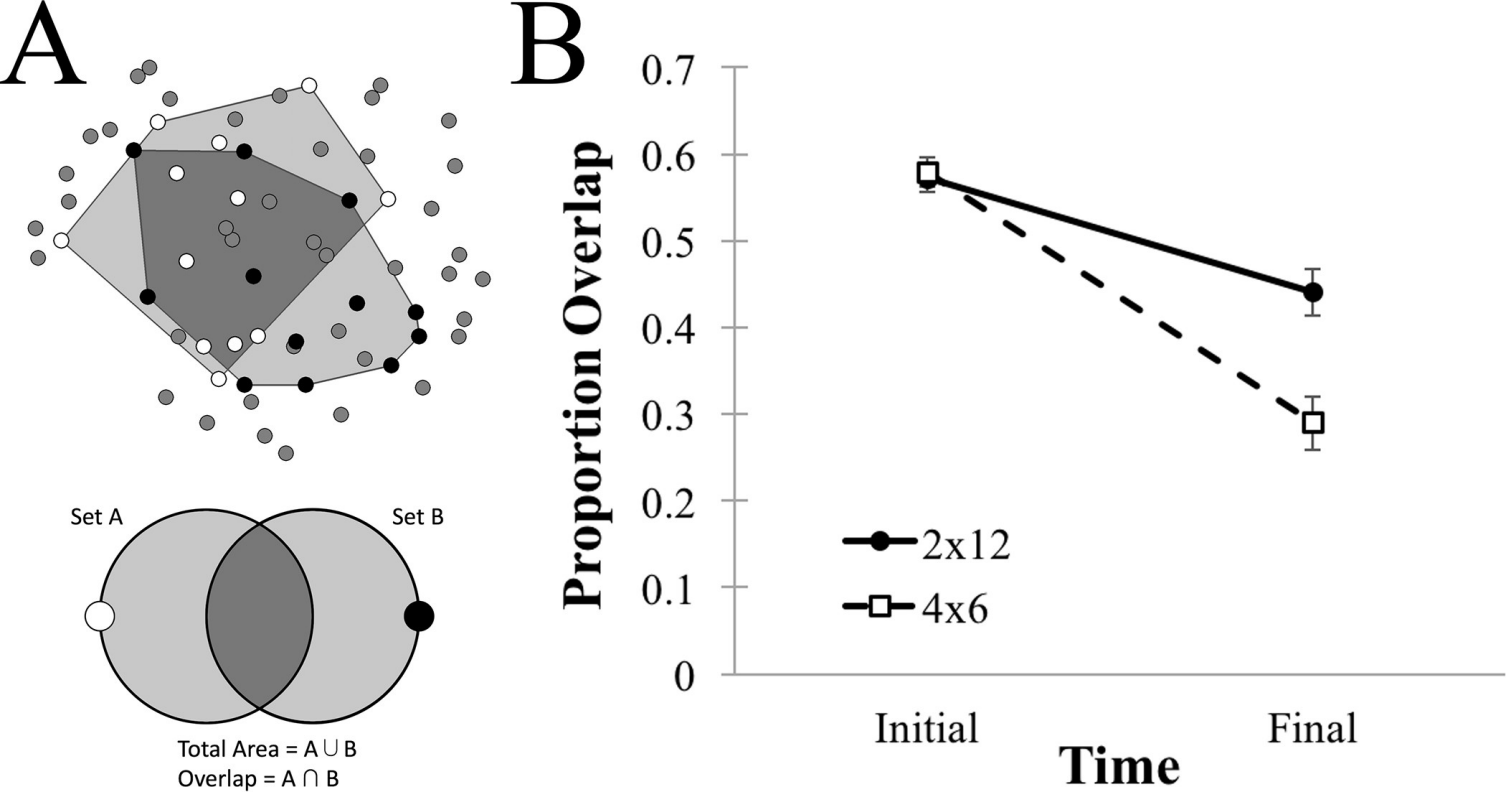
Proportion of overlap between target subsets. **(A)** Example and schematic diagrams of the overlap measure. Proportion overlap is equal to the areal intersection of the subsets divided by the total area of their union. **(B)** Average proportion of overlap for the 2x12 (solid) and 4x6 (dashed) conditions, plotted for Initial and Final configurations, with error bars depicting one standard error of the mean.

Average area reduction shows that participants act to reduce the distance between items generally, but does not indicate if they are sensitive to the different item subsets present in the target stream. To evaluate the degree to which participant-generated organizations reflect the distinction between different subsets, we measured the amount of overlap between subset hulls. In particular, we took the total area covered by all of the hulls, and determined the proportion of that total area that was overlapping between two or more subsets (see Fig 4A). The fact that hull areas decrease tells us only that participants are sensitive to the importance of targets generally (relative to non-targets), but it does not tell us whether the additional community structure within the target set has an influence on organization. However, if participants reduce hull areas as well as separating individual subsets (decreasing the proportion of overlap) then this indicates that the community structure is being mirrored in the spatial organization.

Average proportion overlap is plotted in Fig 4B, and was analyzed with a Time (Initial, Final) by Condition (2x12, 4x6) ANOVA. As with area, the proportion of overlap between subsets was lower for the 4x6 as compared to the 2x12 condition, F(1,97) = 9.12, MSE = .0318, p = .0032, and there was a smaller proportion of overlap for the Final as compared to the Initial configuration, F(1,97) = 94.68, MSE = .0230, p < .0001. In contrast to area, however, the interaction was also significant, F(1,97) = 13.83, MSE = .0230, p = .0003. Although the proportion of overlap did not differ between conditions in the Initial configuration, t(97) = -.1565, p = .8759, overlap was significantly lower for the 4x6 as compared to 2x12 condition in the Final configuration, t(97) = 3.8130, p = .0002. Note that, in both cases, overlap was significantly reduced over time (ts > 4.33, ps < .0001). We can conclude, then, that in both conditions, participants are not only reducing distances between targets in a general way, but they are also separating targets into distinct subsets. This separation tendency, however, appears enhanced for the 4x6 condition relative to the 2x12 condition.

### Performance

We next evaluate whether individual differences in performance on the task are related to differences in the two organizational characteristics reported above. To account for small variations in the total task time, we quantify participant performance in terms of targets obtained per unit time as opposed to the absolute total. Participants completed an average of 10.1 targets per minute (SD 4.56) in the 4x6 condition, and an average of 7.1 targets per minute (SD 2.27) in the 2x12 condition. This difference was highly significant, t(70.14) = 4.04, p = .0001 (degrees of freedom using Satterthwaite’s correction for unequal variance).

**Fig 5:**
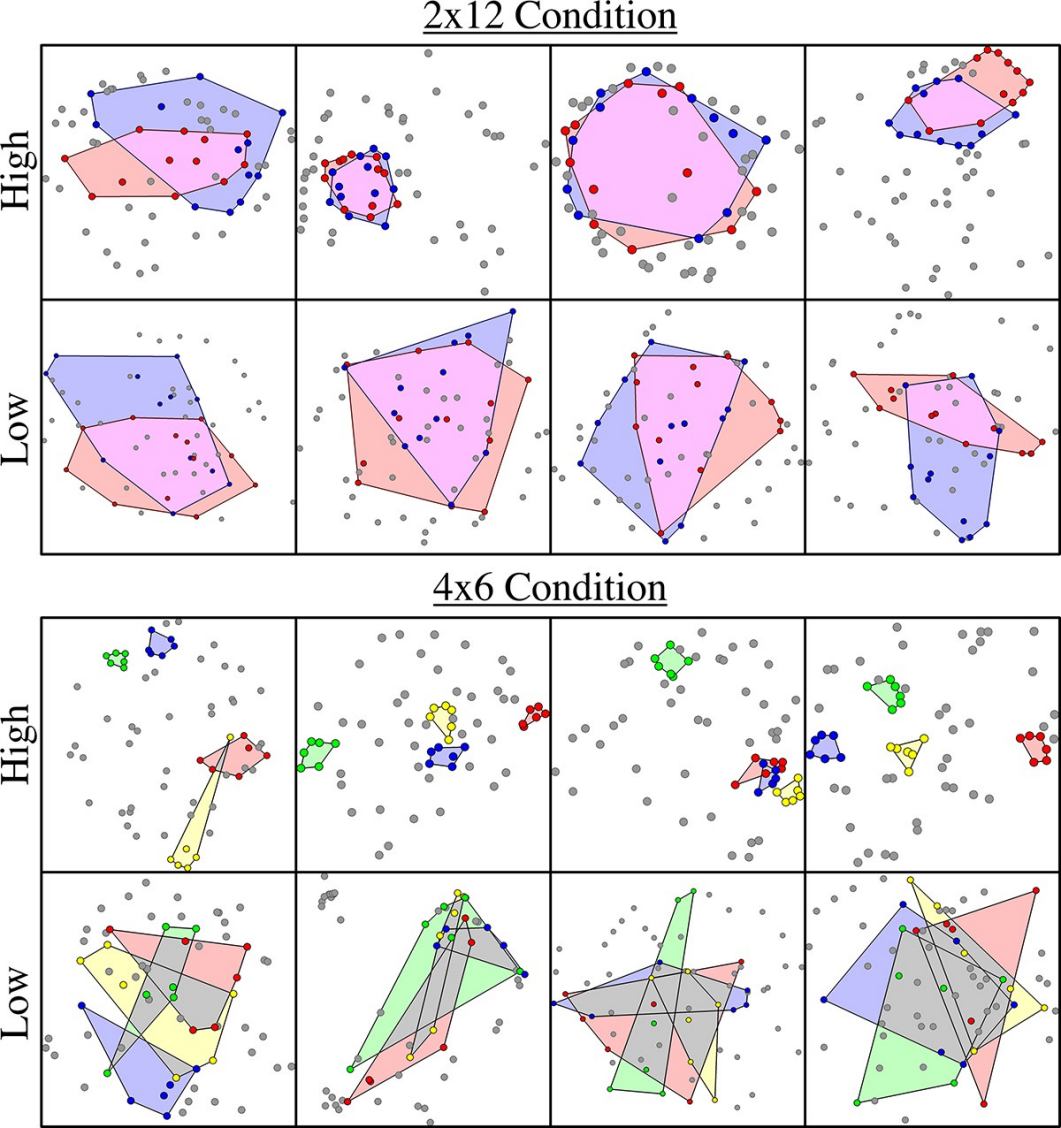
Example terminal configurations for subjects with the highest and lowest performance in each condition (plotted in raw coordinate space; see methods and Fig 2). Target subsets are color-coded and hull areas are shaded (see online version for color figure).

Given the range of target acquisition rates, we can evaluate the extent to which individual differences in the amount of area and overlap reduction might account for differences in performance. In Table 1, we present the results of a linear regression predicting performance on the basis of Final (participant-generated) hull characteristics (area and overlap), including Condition (2x12, 4x6) and all linear interaction terms. For qualitative reference, we show example terminal configurations for high- and low-performance subjects in each condition in Fig 5.

**Table 1:** 

Predictor	B	t	p
*Constant*	*8*.*158*	*4*.*10*	*<* .*001*
Condition*	7.045	3.18	**.*002***
Area	-1.904	-.76	.452
Overlap	0.043	.01	.991
Condition X Area*	-15.762	-2.74	**.*007***
Condition X Overlap*	-10.083	-2.10	**.*038***
Area X Overlap	1.655	.37	.710
Condition X Area X Overlap*	24.620	2.19	**.*031***

* significant at p < .05

The overall model provides a reasonable account of performance, Adj. R^2^ = .408, F(7,91) = 10.65, MSE = 8.842, p < .0001. The significant Condition coefficient recapitulates the observation that performance was overall higher in the 4x6 condition. More interestingly, although absolute area and overlap in the final configuration proved poor predictors on their own, the interactions between these terms and Condition were robust predictors. To clarify these interactions, we conducted two additional regressions–splitting by Condition (Table 2).

**Table 2:** 

	2x12			4x6		
Predictor	B	t	p	B	t	p
*Constant*	*8*.*158*	*5*.*62*	*<* .*001*	*15*.*202*	*12*.*87*	*<* .*001*
Area*	-1.904	-1.04	.305	-17.666	-2.81	**.*007***
Overlap*	.043	.02	.988	-10.040	-2.76	**.*008***
Area X Overlap*	1.655	.51	.611	26.276	2.09	**.*043***

* significant at p < .05

In the 2x12 Condition, the overall model provided a very poor explanation for the data, Adj. R^2^ = .091, F(3,46) = 2.63, MSE = 4.692, p = .061. Indeed, none of the predictors bore any significant relation to performance. For the 4x6 condition, the model was much better, Adj. R^2^ = .371, F(3,45) = 10.42, MSE = 13.083, p < .0001, and both final area and final overlap were significant predictors of performance, as was their interaction. These results indicate that, although at a population level participants in both conditions engaged in generalized organizational behaviors (area and overlap reduction), individual differences in the amount of these behaviors was related to performance only for the 4x6 condition. It is possible, then, that benefits to performance emerge only after a minimum level of basic organization, so that only in the 4x6 condition, where area and overlap were much lower overall, was there a measurable impact on performance. The interaction term is at first surprising, as the coefficient is positive, whereas Area and Overlap, as independent predictors, both have negative coefficients. On closer inspection, the form of the interaction produces a cross-over in the predictive direction of Overlap as a function of Area (or vice versa). In particular, the model suggests that when Area is low, reduced Overlap is beneficial to performance, whereas when Area is high, *increased* overlap is beneficial to performance (estimated transition point ~.382). This is a sensible pattern, however, for although area reduction and concurrent overlap reduction is the more typical and the more effective approach (>65% of participants had final areas below the estimated transition point, and performance for this group was significantly better than for those with final areas above the transition point, t(47) = 3.906, p = .0003), *if* individual set areas remained large, so that each set’s items were broadly distributed, then it would be better to have all of these sets share the *same* extended region of space, in contrast to having several distinct and widely dispersed groups.

### Emergence of organization

Having established that participants in both conditions are engaged in meaningful target-stream-dependent organizational behaviors, we turn next to the question of how and when this organization emerges over the course of the task. Does organization emerge in occasional broad changes to the configuration of many items, or does it emerge through single adjustments here and there whose effects accumulate over time? Relatedly, we ask if organization emerges incidentally through small changes to item positions, or deliberately through large changes to item positions? These two questions address, respectively, the scope and the scale of individual rearrangement events–how many items are involved, and how large are the changes to these items?

To address this question, we identify inter-target intervals with differing levels of area change–a consistent marker of organization–and then evaluate the characteristics of those periods. In this way, we can determine which behaviors are directly associated with increases and decreases to overall organization. To this end, we examine two related measures across levels of Area Change and across Conditions: 1) the number of items moved, and 2) the average distance of these item moves.

#### Area change: Number of items moved

**Fig 6:**
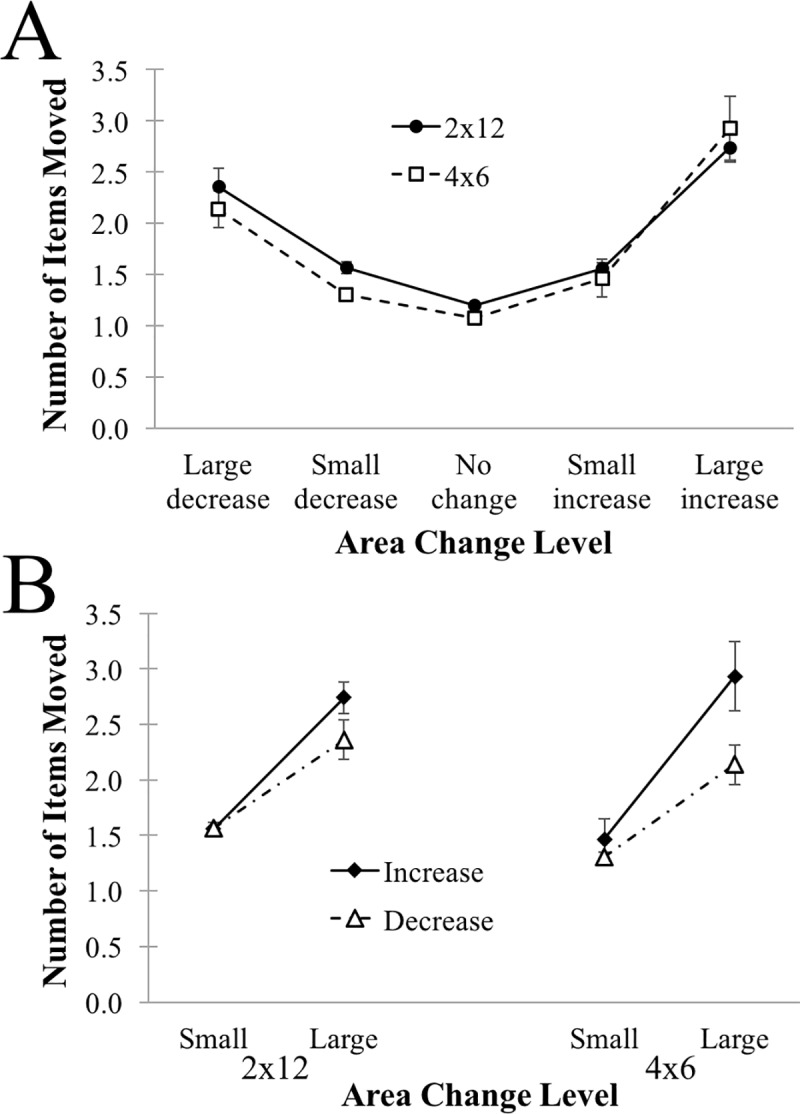
Number of items moved between target acquisitions for 2x12 and 4x6 conditions, plotted as a function of the magnitude of area change occurring in the same inter-target interval. **(A)** Data is plotted across area change levels. **(B)** Data is replotted to clarify the effect of Area Change Level (see text for details). Errors bars depict one standard error of the mean.

We first examine how many items were moved for each of these levels of area change. We computed the cumulative density function for the absolute (i.e., unsigned) change in area (excluding 0, i.e., no change) in each condition, and then obtained the midpoint. Reincorporating sign, this provides a roughly equal sampling of groups: Large Decreases (< = -midpoint), Small Decreases (> -midpoint and < 0), Small Increases (> 0 and < midpoint), and Large Increases (> = midpoint). We are additionally left with a number of No-change periods, which were excluded from the CDF computation. The average number of items moved is plotted in Fig 6A, and was analyzed with a Level (Large Decrease, Small Decrease, No Change, Small Increase, Large Increase) by Condition (4x6, 2x12) ANOVA. We find only the effect of Level reached significance, F(4,387) = 51.08, MSE = .929, p < .0001, characterized by a U-shaped function. Neither the main effect of Condition nor the interaction approached significance (Fs < 1, ps > .383).

To better understand the effect of Level, we reframed the analysis in terms of the direction of the change (Increase, Decrease) and the magnitude of the change (Small, Large), ignoring the no change condition. The data are replotted in these terms in Fig 6B. Analyzed in these terms, we find no effect of Condition, F(1,97) = .31, p = .5763, and no interactions with Condition (Fs < 2.0, ps > .162). There was, however a significant effect of Direction, with more moves for area increases than for area decreases, F(1,97) = 19.99, MSE = .93057, p < .0001, a significant effect of Magnitude, with more moves for large area changes than for small area changes, F(1,97) = 76.77, MSE = 1.91944, p < .0001, and a modest interaction between Direction and Magnitude, F(1,97) = 3.96, MSE = .94383, p = .0494. The interaction was resolved with paired-sample t-tests, revealing no difference between Increases and Decreases when area changes were Small, t(98) = -.9127, p = .3637, but significantly more moves made for Increases than for Decreases when area changes were large, t(98) = -3.6616, p = .0004. On the whole, these results indicate that the key organizational outcome of area reduction appears to emerge incrementally, with only 2–3 items being moved at a time, rather than emerging as a consequence of major multiple-item rearrangements. Indeed, moving larger numbers of items at a time appears to be associated with area *increases*–i.e., towards less efficient arrangements associated with poorer performance. This suggests that participants are likely not acting on a comprehensive model of the task space and its requirements, but instead on relatively local judgments of recent and upcoming needs.

#### Area change: Item move distance

**Fig 7:**
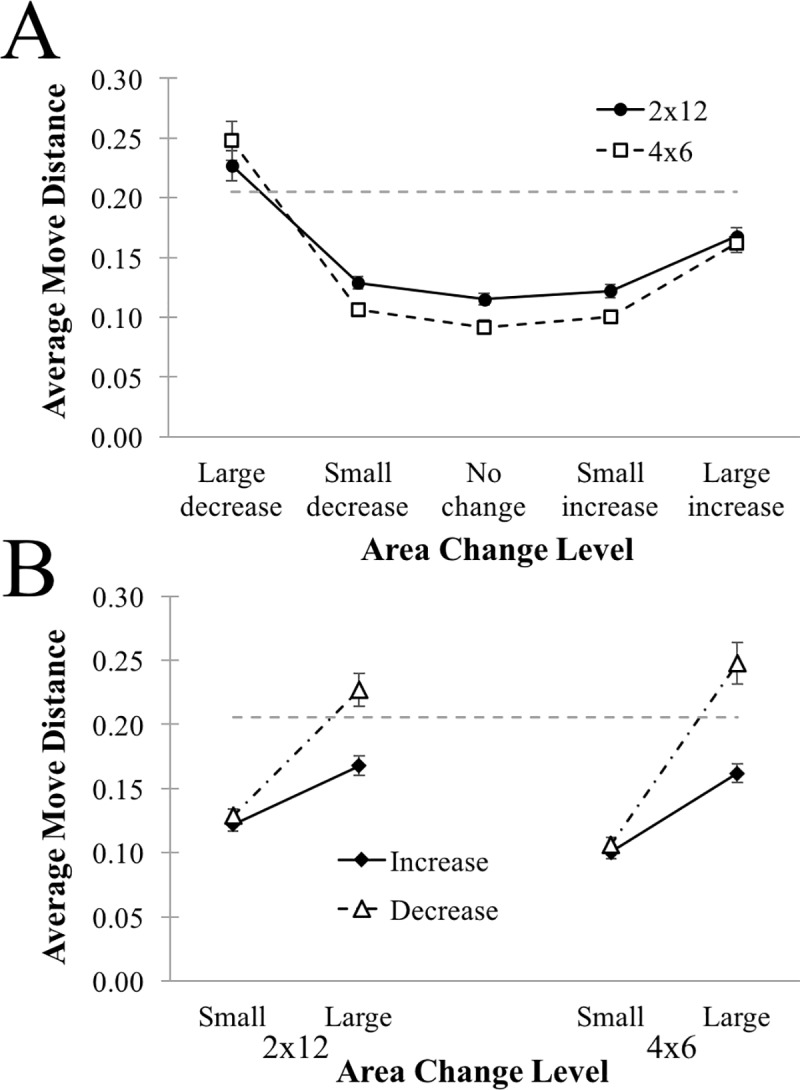
Average distance of item moves between target acquisitions for 2x12 and 4x6 conditions, plotted as a function of the magnitude of area change occurring in the same inter-target interval. **(A)** Data is plotted across area change levels. **(B)** Data is replotted to clarify the effect of Area Change Level (see text for details). Dashed gray line in both plots indicates the radius of the reach area–moves beyond this area required movement in the space. Errors bars depict one standard error of the mean.

We next evaluated the average *distance* of each item moved during periods having differing levels of area change. These data are plotted in Fig 7A, and were analyzed with a Level (Large Decrease, Small Decrease, No Change, Small Increase, Large Increase) by Condition (4x6, 2x12) ANOVA. As with the number of items moved, there was a significant effect of Level on the average distance of item moves, F(4,387) = 101.46, MSE = .00306, p < .0001, characterized again by a U-shaped function. There was no main effect of Condition, F(1,97) = 2.71, p = .103, but there was a modest interaction, F(4,387) = 2.90, MSE = .00306, p = .022. Comparing the 4x6 to the 2x12 condition at each area change Level, we find no difference between conditions for large increases or large decreases (ts < 1.02, ps > 3.14), and slightly longer average move distances for 2x12 than for 4x6 at each of the remaining Levels (i.e., for small changes and no change; all ts > 2.836, ps < .0056).

We again reframed the data in terms of the Size and the Direction of the area change, and this data is plotted in Fig 7B. We find no main effect of Condition, F(1,97) = 1.05, p = .3075, and no interactions with Condition (largest F = 3.57, p = .0617). Moves were typically shorter for area Increases than for area Decreases, F(1,97) = 53.09, MSE = .00276, p < .0001, and were shorter for Small area changes than for Large area changes, F(1,97) = 133.75, MSE = .00688, p < .0001. The interaction between Size and Direction was also significant, F(1,97) = 62.01, MSE = .00244, p < .0001. Average item moves were shorter for area Increases than for area Decreases both when area changes were Small, t(98) = 2.8254, p = .0057, and when area changes were Large, t(98) = 7.6954, p < .0001 –but the magnitude of this difference was significantly greater for Large area changes (Decrease—Increase = .0725) than for Small area changes (Decrease—Increase = .0063): t(98) = 7.1067, p < .0001.

We perform an additional task-specific analysis on average item move distances, comparing them to the reach radius (shown in Fig 7 as a dashed grey line), noting that items moved beyond the reach radius are categorically different from those within the radius–as they require that the agent also moves within the space. Moves beyond the reach radius, then, can be more explicitly identified as deliberate rather than incidental rearrangements. We find that, for Large Decreases exclusively, the average item move distance exceeded the reach radius, t(98) = 3.132, p = .0023. For all other area change levels, the average item move distance was smaller than the reach radius (all ts < -7.752, ps < .0001). This indicates that Large Decreases in area are uniquely characterized by item moves exceeding the reach radius. Taken together with the results for the number of items moved, we arrive at a clear picture of the typical process of organization: large improvements to organization (reductions in hull area) arise from deliberate long-distance movement of a small number of items. In other words, organization is incremental rather than global, but is clearly deliberate rather than incidental or emergent. These observations provide important constraints for understanding how individuals organize space, and predicting which kinds of task structures participants should be best able to adapt their environment to.

## Discussion

The present work stems from a simple but important observation: that human beings are active observers with meaningful control over their environments. Consistent with prior work, we find that unprompted organization is a robust behavior, even in the relatively abstract and limited context of the present experiments–when given the opportunity, participants will restructure the environment to facilitate their ongoing and upcoming pragmatic actions. This most basic observation warrants emphasis. Although there are many successful theories of attention and perception, there is an inevitable disconnect from real world behavior to the extent that these models do not provide an observer with agency. The role of clutter in disrupting search or object identification, for example, is important only to the extent that clutter is actually present in the real world. Without understanding how objects come to be arranged in real environments, it is difficult to know which perceptual factors are of greatest importance in predicting naturalistic behavior.

The limited existing work provides some insight into these processes–for example, showing that participants actively reduce clutter by peripheralizing low-usage objects, and contracting the distance between high-usage objects [27]. In the present work, we extend these results, showing that in addition to contraction and isolation of target objects, participants show an organizational tendency to partition the environment into distinct functionally-related subsets of objects. These two behaviors both serve to reduce the expected cost of searching for and accessing successive targets, and indeed, both contraction and partitioning independently predict performance.

Importantly, we also show that these tendencies differ as a function of the specific task-structure. In particular, segregation appears favored when the task-environment consists of many small groups of related objects (4x6 condition) as compared to fewer large groups of related objects (2x12 condition). Additionally, we find that the relation between task performance and organization is also dependent on the statistics of the target sequence. While individual differences in both area reduction and segmentation independently predicted performance in the 4x6 condition, neither organizational variable predicted performance in the 2x12 condition.

One possible explanation for this difference is that expected transition rates for items within and between subsets are too subtle in the 2x12 condition for participants to reliably partition the two sets. Indeed, although expected across-set transition rates are similar for the two conditions (.0033 vs .0049 for 4x6 and 2x12, respectively), expected within-set transition rates are more than doubled for the 4x6 condition relative to the 2x12 condition (.1883 vs .0856, respectively). Comparing the two rates, we find that in the 4x6 condition, pairwise transitions within-set are ~57 times more likely than pairwise transitions across sets, whereas in the 2x12 condition, they are only ~17.5 times more likely. This stronger set partitioning may have allowed participants in the 4x6 condition to move beyond generalized organizational tendencies into a range where measurable performance differences could arise. This notion is further supported by the observation that organization emerges over time through temporally-local, rather than global changes–i.e., typical organization-improving actions are characterized by only moving a small number of individual items at a time. In this case, we might expect that local item-item statistics would be the strongest driver for organizational decisions, and that the weaker signal in the 2x12 condition may have been insufficient to guide these individual placement decisions. It is also possible that participants in the 2x12 condition were aware of the distinction between sets, but that the perceived advantages of segregation were not high enough to warrant the effort. Indeed, it is not unreasonable to suggest that participants have an easier time holding six positions in working memory rather than twelve. Consequently, partitioning into groups of six may have cognitive advantages, where partitioning into groups of twelve does not. Ultimately, we expect the ‘optimal’ partitioning to reflect tradeoffs between the strength of the grouping signal (within vs between set transition rates), the memory burden of representing individual group locations, and the memory burden of representing individual item locations within each group. Future studies examining a broader range of target sequence statistics will be important for clarifying these possibilities.

In addition to measuring the organizational outcomes at the conclusion of the study, we also examined how behaviors throughout the task produced these outcomes. We report that the most functional organization changes–i.e., those leading to the largest reductions in inter-item distances–arose from moving a relatively small number of items over a relatively large distance. This is in contrast to periods of counterproductive organization–i.e., those leading to the largest *increases* in inter-item distances–which resulted from movement of more items over shorter distances. For our participants, then, the most effective organization seemed to arise from deliberate but limited adjustments to the display configuration. This behavior does not fall neatly into either of the categories we predicted at the outset (i.e., gradual emergent organization or global deliberate organization), but instead seems to straddle the line between these possibilities. Sequentially related items did not gradually drift together over time through incidental replacement decisions, but were instead very deliberately moved together. However, these deliberate adjustments were not completed en masse according to a global plan or strategy, but instead arose on an apparently case-by-case basis, with organization interspersed with task progression.

### Relation to search and spatial memory

We have focused our discussion here on organizational strategies, but it is clear that both search and memory processes are integral to the current task. Although it is beyond the scope of the current work to fully integrate these three subjects, we wish to highlight several particularly relevant features. First, we note that a range of spatial memory tasks (e.g., [29–33]) have shown the importance of clustering–both in time (i.e., for items studied in sequence), and in space (i.e., for items nearby at study). Similarly, spatial memory is improved when sequences follow a clear spatial structure [34–37], and when comparing within-group to between-group comparisons [38]. Although we did not explicitly test participant memory in the present tasks, it would be valuable to determine in future work whether individual differences in organization would be reflected in clustering tendencies during recall.

In more direct relation to the current task, we note that human observers are also very good at remembering the locations of targets that have been previously *searched* for, absent explicit instructions to memorize, with indirect evidence arising from decreased search times and accurate early search trajectories (e.g., [39,40]), and direct evidence arising from explicit memory tests–for example, participants in a study by Solman & Kingstone [41] had spatial memory accuracies in the range of 80–90% for 48 distinct items searched for only 5 times each. On this basis, we might expect that the role of traditional search in the present task is likely to decline rapidly over time as individuals grow familiar with the environment’s arrangement. On the other hand, these prior results arise from repeated exposure to *static* displays, and so the impact of continual small adjustments to the configuration is unclear. In one telling result, participants completed a repeated search task where the search items were made to ‘drift’ by a small amount on each successive trial–and the usual benefits of repetition were almost completely suppressed [39].

Based on the evident capacity for accurate memory, but also its apparent fragility in the face of disruptions, it is possible that participants in the present task could be reluctant to rearrange the display excessively, as the potential disruption to memory might outweigh the potential benefits of reorganization. There is some support for this account in prior work on prgamatic action. In particular, we note that in Zhu and Risko’s [26] pen-placement study, participants were likely to rearrange the position of the pens only when one of the pen-holders was significantly costlier to access than the other. When the holders differed more modestly, participants often simply left the pens as they first found them–preferring habit and continuity over a strictly optimal configuration for physical effort reduction. A similar calculus of convenience and familiarity may underlie the behaviors reported here, and may place important constraints on the extent to which we should expect ‘optimality’ in real organizational decisions. In particular, it may be critical to properly quantify and account for the cost and utility of existing memories when considering the potential advantages of an organization decision.

## Conclusions

Naturalistic tasks require engagement with the physical world in addition to cognitive processing. Past work has emphasized behaviors that take advantage of this physicality to ‘offload’ cognitive demands onto the world [20], for example by writing information down instead of memorizing it [42], or by placing cues in the world to remind oneself of a necessary future action [12,16]. Relatively little work has considered how the environment can be manipulated to reduce the pragmatic, non-cognitive costs involved in locating and accessing necessary tools and resources for a multitude of ongoing tasks on expected future tasks.

Here we demonstrate robust pragmatic organization that facilitates ongoing actions, and reflects sensitivity to long-term patterns in demands. In particular, we find reorganizing space to bring task-relevant items closer together, while simultaneously drawing apart items from distinct sub-tasks. We further report that these organizational tendencies, and their relationship to performance, vary depending on the underlying statistical features of the task–specifically, the number and size of the subsets within a community structure. Whether these differences reflect limitations in learning or in planning remains an open question. Finally, we report that the most functionally useful organizational changes (i.e., those leading to contraction of related subsets) arise from a small number of long-distance object movements, in contrast to counterproductive reorganizations which involve a greater number of short-distance object movements. This suggests that spatial organization, in the present context, is best understood as a deliberate but incremental process. Collectively, these observations provide new insight into the nature of human organization, and inform related theories of routine action, planning, and metacognition. We argue the observed organizational behaviours can be understood as long-term pragmatic actions–actions that are neither explicitly driven by the immediate needs of the task, nor directly related to reducing cognitive burdens, but instead are helpful in reducing the expected effort for both current and future predicted needs.
